# Automated analysis of rabbit knee calcified cartilage morphology using micro‐computed tomography and deep learning

**DOI:** 10.1111/joa.13435

**Published:** 2021-03-29

**Authors:** Santeri J. O. Rytky, Lingwei Huang, Petri Tanska, Aleksei Tiulpin, Egor Panfilov, Walter Herzog, Rami K. Korhonen, Simo Saarakkala, Mikko A. J. Finnilä

**Affiliations:** ^1^ Research Unit of Medical Imaging, Physics and Technology University of Oulu Oulu Finland; ^2^ Department of Applied Physics University of Eastern Finland Kuopio Finland; ^3^ Department of Diagnostic Radiology Oulu University Hospital Oulu Finland; ^4^ Ailean Technologies Oy Oulu Finland; ^5^ Human Performance Laboratory Faculty of Kinesiology University of Calgary Calgary AB Canada

**Keywords:** animal models, bone, histology, osteoarthritis, segmentation

## Abstract

Structural dynamics of calcified cartilage (CC) are poorly understood. Conventionally, CC structure is analyzed using histological sections. Micro‐computed tomography (µCT) allows for three‐dimensional (3D) imaging of mineralized tissues; however, the segmentation between bone and mineralized cartilage is challenging. Here, we present state‐of‐the‐art deep learning segmentation for µCT images to assess 3D CC morphology. The sample includes 16 knees from 12 New Zealand White rabbits dissected into osteochondral samples from six anatomical regions: lateral and medial femoral condyles, lateral and medial tibial plateaus, femoral groove, and patella (*n* = 96). The samples were imaged with µCT and processed for conventional histology. Manually segmented CC from the images was used to train segmentation models with different encoder–decoder architectures. The models with the greatest out‐of‐fold evaluation Dice score were selected. CC thickness was compared across 24 regions, co‐registered between the imaging modalities using Pearson correlation and Bland–Altman analyses. Finally, the anatomical CC thickness variation was assessed via a Linear Mixed Model analysis. The best segmentation models yielded average Dice of 0.891 and 0.807 for histology and µCT segmentation, respectively. The correlation between the co‐registered regions was strong (*r* = 0.897, bias = 21.9 µm, standard deviation = 21.5 µm). Finally, both methods could separate the CC thickness between the patella, femoral, and tibial regions (*p* < 0.001). As a conclusion, the proposed µCT analysis allows for ex vivo 3D assessment of CC morphology. We demonstrated the biomedical relevance of the method by quantifying CC thickness in different anatomical regions with a varying mean thickness. CC was thickest in the patella and thinnest in the tibial plateau. Our method is relatively straightforward to implement into standard µCT analysis pipelines, allowing the analysis of CC morphology. In future research, µCT imaging might be preferable to histology, especially when analyzing dynamic changes in cartilage mineralization. It could also provide further understanding of 3D morphological changes that may occur in mineralized cartilage, such as thickening of the subchondral plate in osteoarthritis and other joint diseases.

## Graphical abstract

We present a micro‐computed tomography‐based method with deep learning segmentation for analyzing calcified cartilage (CC) thickness. The comparison of regions co‐registered to histology yielded a strong Pearson correlation (*r* = 0.90). Both methods were able to separate the CC thickness properties between tibia, femur, and patella.

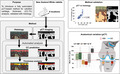

## 1 INTRODUCTION

Calcified cartilage (CC) is a mineralized tissue delineated from the non‐calcified articular cartilage by the tidemark, and from the subchondral bone by the cement line (Madry et al., 2010). The CC has an important role in anchoring the articular cartilage to the subchondral bone via individual collagen fibrils (Sophia Fox et al., 2009). For healthy conditions, the relative CC thickness (CC.Th) to the total cartilage is nearly constant, but the CC volume relative to the total cartilage volume varies and has been shown to range from 3.23% to 8.8% (Müller‐Gerbl et al., 1987). Blood vessels from the subchondral bone extend into the CC layer, providing nutrients to the local chondrocytes (Madry et al., 2010). Furthermore, based on the current literature, CC is a dynamic tissue undergoing changes with mechanical loading, ageing, and joint pathology, e.g. osteoarthritis (Hoemann et al., 2012).

The thickness of articular cartilage (Cohen et al., 1999; Kiviranta, Tammi, et al., 1987) and subchondral bone (Milz & Putz, 1994) vary greatly in different areas of the knee joint with a high thickness in heavily loaded areas. It can be hypothesized that similar changes are present in the CC as well. An early study on CC.Th revealed regional differences within the human femoral head (Müller‐Gerbl et al., 1987). Furthermore, clear regional differences in equine CC have been reported (Kim et al., 2013; Martinelli et al., 2002). By contrast, in canine knees, only minor regional differences have been found (Kiviranta, Tammi, et al., 1987). These differences related to anatomical location could be linked to the local loading environment.

In general, exercise and loading are thought to affect the CC structure. The intensity of exercise on heavily loaded joint regions is associated with thicker CC in equine tarsi (Tranquille et al., 2009) and carpus, even without changes in the overlying non‐CC (Murray et al., 1999). An increase in the canine CC.Th was observed with high‐intensity exercise (Oettmeier et al., 1992). By contrast, unloading of knees with immobilization resulted in thinner CC in canine knees (Kiviranta, Jurvelin, et al., 1987). In the human knee joint, similar findings have been reported; both articular and CC are thick in load‐bearing areas and thin under the menisci of the knee (Thambyah et al., 2006).

Two competing events occur in ageing CC: calcification of the deep articular cartilage via advancement of the tidemark (Havelka et al., 1984) and endochondral ossification (bone replacing CC at the cement line) (Doube et al., 2007). The latter is likely dominant as ageing accelerates the thinning of CC and increases the number of tidemarks (Doube et al., 2007; Lane & Bullough, 1980). Although CC.Th varies across humans and different animal species (Frisbie et al., 2006), similar changes in ageing CC have been found in animal models. Thinning of CC, increases in vessel invasion (Pan et al., 2012), as well as chondrocyte apoptosis (Adams & Horton, 1998) have been reported in murine CC with ageing. On the other hand, Murray et al. reported an age‐related increase in CC.Th in the equine tarsometatarsal joint (Murray et al., 2009). Joint pathology can also induce tissue responses in the CC. Remodelling of CC (Doube et al., 2007; Lane & Bullough, 1980) occurs during OA progression, contributing to a decrease in articular cartilage thickness (Goldring & Goldring, 2007). Microfractures in the CC, subchondral bone plate, and the trabeculae lead to the formation of cysts and channels, thereby affecting the cross‐talk between articular cartilage and subchondral bone (Madry et al., 2010).

Traditionally, CC imaging has been performed on images obtained from histological sections (Müller‐Gerbl et al., 1987) as well as backscattered scanning electron microscopy (SEM) in equine (Doube et al., 2007) and human joints (Ferguson et al., 2003; Gupta et al., 2005). Both histology and SEM require extensive and time‐consuming sample processing protocols and allow for two‐dimensional (2D) imaging only. Nowadays, three‐dimensional (3D) volumetric reconstruction of histological (Gerstenfeld et al., 2006) and SEM images (Guo et al., 2014) is possible with serial sectioning and imaging, but the associated processing is laborious and has the potential to introduce errors.

Micro‐computed tomography (µCT) has been widely used to characterize 3D morphology at the micron level, including CC (Kerckhofs et al., 2012; Mehadji et al., 2019). In contrast to histology and SEM, only minimal sample processing is required in µCT. We showed previously that µCT images of the human subchondral plate contain both the mineralized CC and the subchondral bone (Finnilä et al., 2017). Indeed, CC cannot be separated from bone with low‐resolution µCT imaging but becomes visible only in high‐resolution µCT images (Rytky et al., 2020). However, because of the very minor difference in mineralization between the subchondral bone and CC, it is challenging to delineate the interface between CC and subchondral bone also in high‐resolution µCT imaging.

The identification of the tidemark and cement line from µCT images is often conducted manually by researchers. This is a subjective and highly time‐consuming endeavour, especially for tissues with complex shapes. Deep convolutional neural networks (CNNs) have recently shown great promise for automating various segmentation problems. U‐Net (Ronneberger et al., 2015) has been the most popular segmentation architecture for biomedical images in recent years, and it has also been applied to µCT data (Tiulpin et al., 2020). However, the newly introduced Feature Pyramid Networks (FPN) allow for capturing both low‐resolution global features as well as high‐resolution local features at a low computational cost (Lin et al., 2017). Conventional training of CNNs is conducted by initializing the coefficients from a random distribution. An alternative training approach is transfer learning, in which the network is initialized from an existing model, often pre‐trained on ImageNet dataset (J. Deng et al., 2009; Ng et al., 2015). Notably, such an approach works efficiently across domains beyond natural images (Shin et al., 2016; Tiulpin & Saarakkala, 2020). For example, transfer learning from deep residual networks (He et al., 2016) has been used to classify pulmonary nodules from CT images (Nibali et al., 2017), or segment the lungs in chest X‐rays (Solovyev et al., 2020).

In this study, we propose an accurate framework for automated µCT‐based evaluation of the CC.Th in 3D. This requires introducing state‐of‐the‐art deep learning architectures for CC segmentation. To demonstrate the validity of the method, we perform direct comparisons of CC.Th between µCT and conventional histology. We utilized osteochondral samples of New Zealand White rabbits, a frequently used animal model for various musculoskeletal diseases. Furthermore, we hypothesize that the CC.Th varies in different anatomical locations of the knee. We demonstrate the capability of our automatic framework by assessing differences in CC.Th between the different anatomical locations.

## 2 MATERIALS AND METHODS

### 2.1 Sample collection

Sixteen knees were collected from twelve healthy, skeletally mature female New Zealand White rabbits (strain 052 CR). Eight knees were collected from four rabbits (age: 14 months) and eight knees from eight rabbits (age: 12.5 months). Each knee was dissected and divided into six anatomical regions: lateral and medial femoral condyle, lateral and medial tibial plateau, femoral groove, and patella (*n* = 96, Table 1). Details on animal housing, husbandry conditions, and diet are described in a previous study (Mustonen et al., 2019). All experiments were carried out under the guidelines of the Canadian Council on Animal Care and were approved by the committee on Animal Ethics at the University of Calgary (Renewal 3 for ACC Study #AC11­0035).

**TABLE 1:** Descriptive statistics of the rabbits used in the study. On the right, the number of images and samples (separated by/mark) segmented manually is described. These segmentations are used as training data for the deep learning models

# animals	# knees	# Samples	# Histology slices	Manual segmentations	
				Histology	µCT
12	16	96	3/sample	253/87	1050/60

Abbreviation: µCT, micro‐computed tomography.

### 2.2 Imaging

The dissected osteochondral samples were formalin‐fixed. Prior to imaging, samples were wrapped in moist paper, and placed in plastic vials (Cryo.s™) for positional stability. The samples were subsequently imaged using a desktop µCT scanner (Skyscan 1272; Bruker microCT) with a tube voltage of 50 kV, current of 200 µA, and a 0.5 mm aluminum filter. The scanning was conducted in a step of 0.2° over 360° and finally, 1800 projection images with an isotropic pixel size of 3.2 µm were obtained.

The images were reconstructed using the manufacturer's software (NRecon, version 1.7.0.4, beam hardening correction applied). A narrow window with attenuation coefficients 0.085–0.141 mm^−1^ was used to provide high contrast between the bone and CC. The volumes‐of‐interest (VOI) of all samples were selected from the central load‐bearing area (VOI size = 2 mm × 2 mm × sample height). This selection reduced the µCT image stacks to a reasonable size (from ~12 GB to ~700 MB per sample) for the subsequent analysis. See Figure S1 for examples of the preprocessing steps.

After the µCT imaging, samples were prepared for histological analysis. Samples were decalcified using a standard protocol with ethylenediaminetetraacetic acid solution, paraffin‐embedded, and cut into 5‐µm‐thick sections using a microtome (three sections from each region). The sections were stained with Masson‐Goldner's trichrome for identification of the CC layer and imaged using a light microscope (Axioimager 2; Carl Zeiss MicroImaging Gmbh; control software = AxioVision; resolution = 2.56 µm). A total of 281 sections were used in this study.

### 2.3 Training CC segmentation models

**FIGURE 1:**
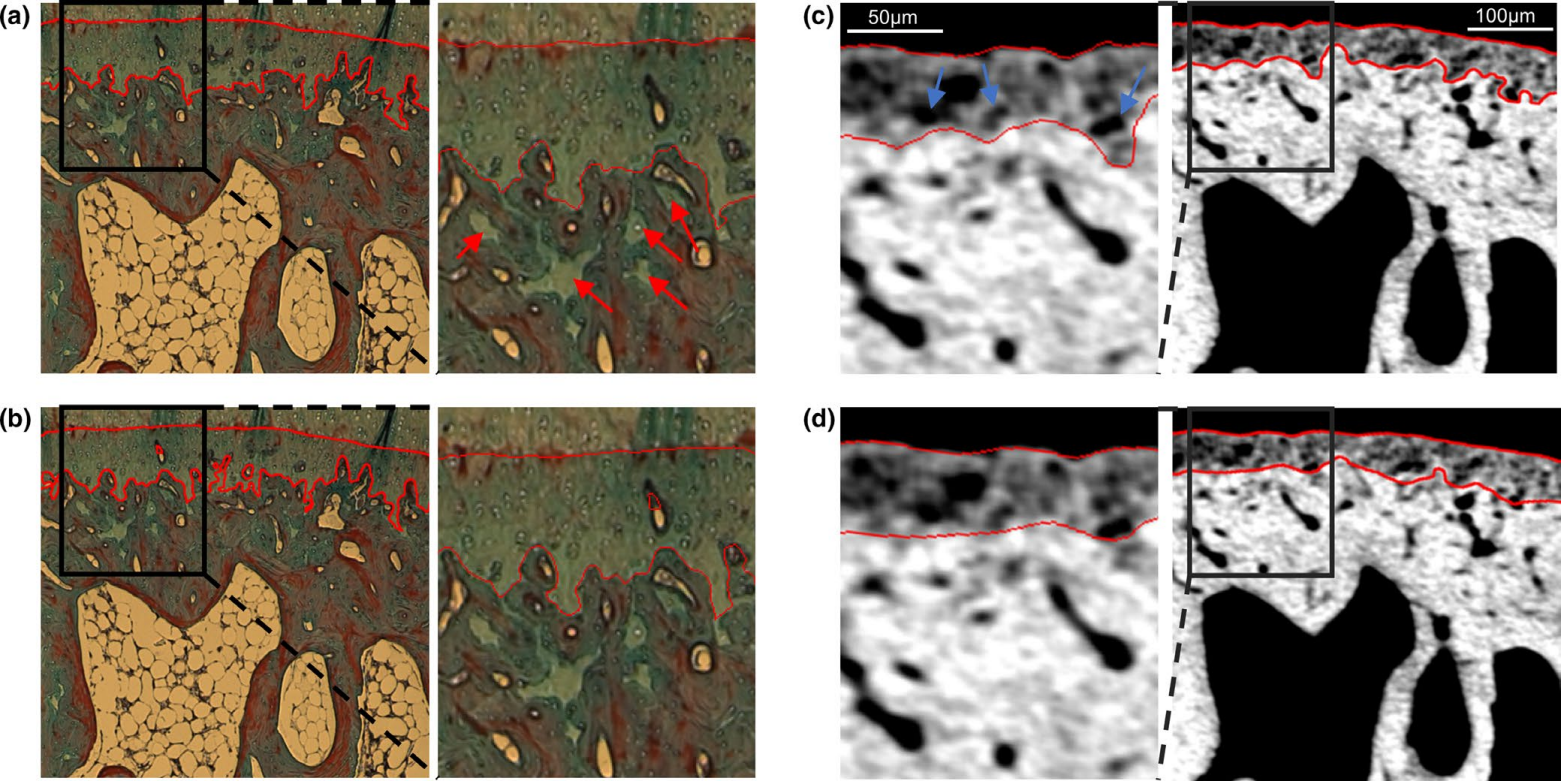
A histological section from the rabbit femoral condyle segmented manually (a) and automatically with the neural network (b). Co‐registered µCT image from the same region with manual (c) and automatic (d) segmentation. Magnified images are provided to allow detailed comparison of the segmentation boundaries. Scale bars for 100 and 50 µm (magnification) are shown in the corresponding images. The blue arrows refer to chondrocytes inside the CC layer. Isolated small areas of CC are excluded from the manual segmentations (red arrows). Based on the magnified images, the deep CC layers seen in histology are not observed with µCT, leading to possible overestimation of CC.Th in histology. µCT, micro‐computed tomography; CC, calcified cartilage

A total of 253 histology images were segmented manually from 87 osteochondral samples. The boundaries of the CC were drawn based on the distinct collagen staining of CC compared to the articular cartilage and subchondral bone plate. At the interface between CC and articular cartilage, the topmost tidemark was followed. The discrimination was also supported by the higher staining intensity of Aniline blue in CC. Subchondral bone has the highest Aniline blue intensity and guides the segmentation at the complex interface between CC and subchondral bone. However, narrow CC cavities (>10 pixels) and small isolated areas that are not connected to the CC layer were excluded (Figure 1a, red arrows). To limit the time required for manual segmentation, smaller regions were segmented from the full histology images (approximately one third of a full histology section). For the µCT, manual annotations were conducted for 60 samples from 10 knees according to two inclusion criteria: (1) a CC region with a distinct grayscale gradient and (2) the presence of chondrocytes inside the CC layer (Figure 1c, blue arrows). Annotations were done for 10–30 slices per sample, evenly spaced within each volume resulting in a total of 1050 annotated slices. The manual annotations were used as the gold standard for the automated segmentation algorithms and for conducting a reference analysis for the CC morphology.

The fully automatic CC segmentation was conducted using a deep learning pipeline inspired by Solovyev et al. (2020) on Python 3.7. The pipeline was built using an in‐house developed *Collagen*‐framework (https://github.com/MIPT‐Oulu/Collagen). For the histology segmentation, we used ResNet‐34 (He et al., 2016) pre‐trained on ImageNet (J. Deng et al., 2009). We used a U‐Net decoder with batch normalization in this model. The network was trained for 100 epochs under fourfold cross‐validation, splitting the training and validation folds with respect to rabbit ID. For the µCT segmentation, we used ResNet‐18 as our base model, and also an FPN decoder, which had instance normalization as well as the spatial dropout. Briefly, the normalization reduces bias for individual features with large values, whereas dropout reduces model overfitting by zeroing random nodes of the network. This model was also trained in fourfold cross‐validation but for 60 epochs due to faster convergence.

**FIGURE 2:**
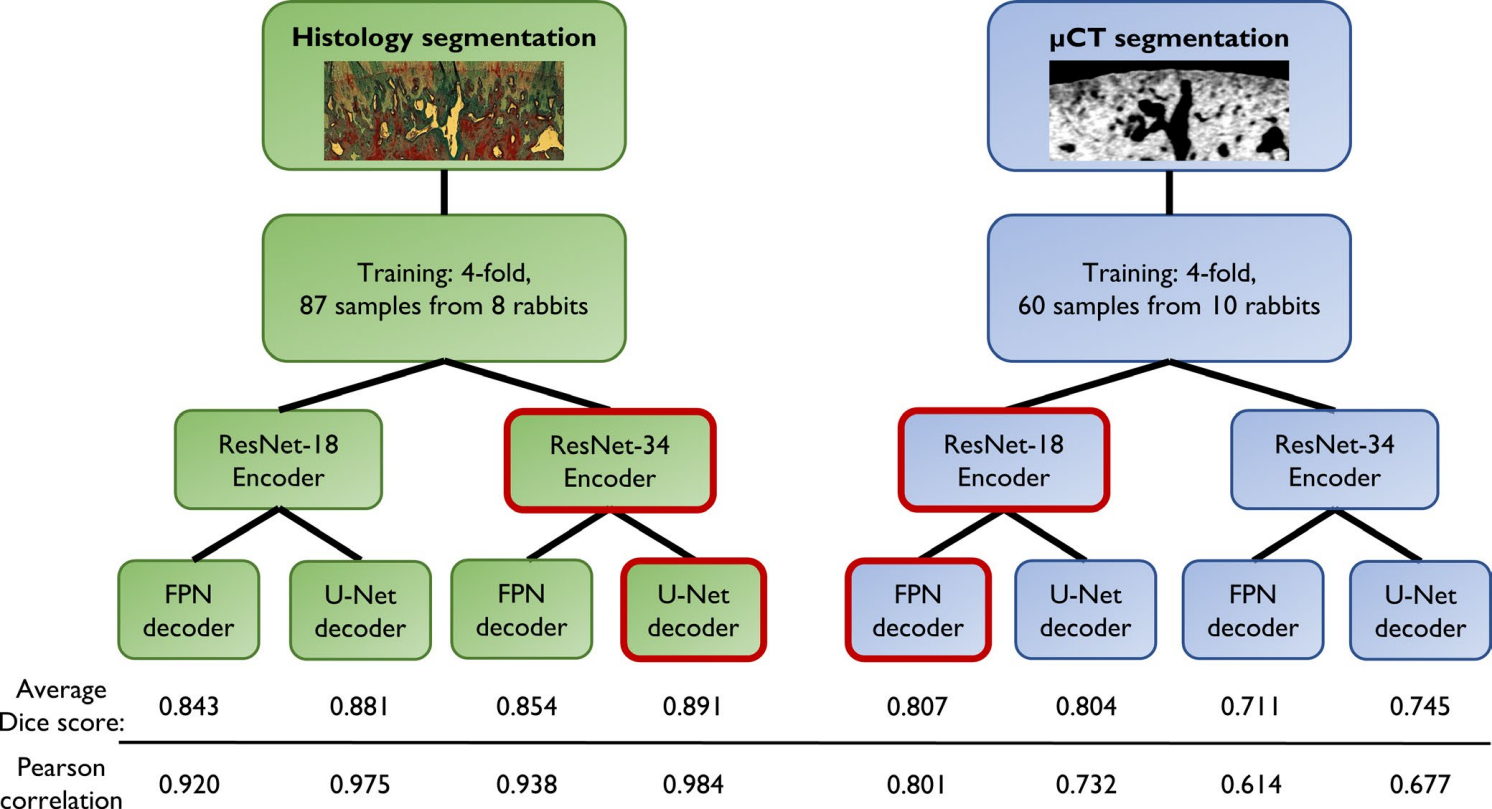
Illustration of the model training process. For both histology‐ and µCT segmentation, a total of four models were trained with two different encoder and decoder designs. Based on the experiments, ResNet‐34 and U‐Net were more suitable for the complex histology masks (Dice score = 0.891), whereas ResNet‐18 and FPN yielded higher performance for the smoother µCT masks (Dice score = 0.807). Pearson correlation of the subsequent CC.Th analysis (bottom row) supported the choice of the segmentation models. µCT, micro‐computed tomography; CC, calcified cartilage; FPN, Feature Pyramid Networks

We used a combination of binary cross‐entropy and soft Jaccard index as the optimization loss function. Binary cross‐entropy is one of the most popular segmentation metrics and can result in stable convergence. However, Jaccard index can account for class imbalance, such as an imbalance between the CC and the surrounding tissue. To facilitate a robust segmentation model, we used several image augmentation techniques (Table S1) from the SOLT library (https://github.com/MIPT‐Oulu/solt) to diversify the training data. To assess the final segmentation performance, we calculated the loss and Dice score coefficient as an average from the evaluation folds. The selection of the encoder and decoder was done based on an ablation study (Figure 2; Figure S2).

### 2.4 Model application on new images (inference)

During inference, CC was predicted for the full histology images, by combining smaller tiles with a sliding window (512 × 1024 ‐pixel window with 256 × 512 ‐pixel steps), averaging the overlapping predictions. The tiling was used to avoid memory issues on the graphical processing unit while segmenting larger areas of CC. The tiles were combined, averaging the overlapping areas and predictions from every fold. Subsequently, a threshold was applied to the prediction map by using a probability of 0.8 (a high threshold was used for the exclusion of ambiguous areas from the maps, especially for the µCT images). In the case of the µCT stacks, the inference was conducted slice‐by‐slice with similar tiling. The predictions were averaged from every fold as well as the coronal and sagittal planes for obtaining the final probability map.

The histology masks were post‐processed by removing regions smaller than 500 pixels. This ensured the removal of small artefacts while retaining large CC regions that could be disconnected due to a fold in the histology section (Figure S3). In the µCT post‐processing, masks were subjected to a sweep operation to keep only the largest object. This ensured the removal of possible false positives occurring on the tiles far from the actual CC layer. Finally, all CC masks were median filtered with a radius of 12 pixels (3D filtering in the case of µCT).

### 2.5 Morphological analysis

**FIGURE 3:**
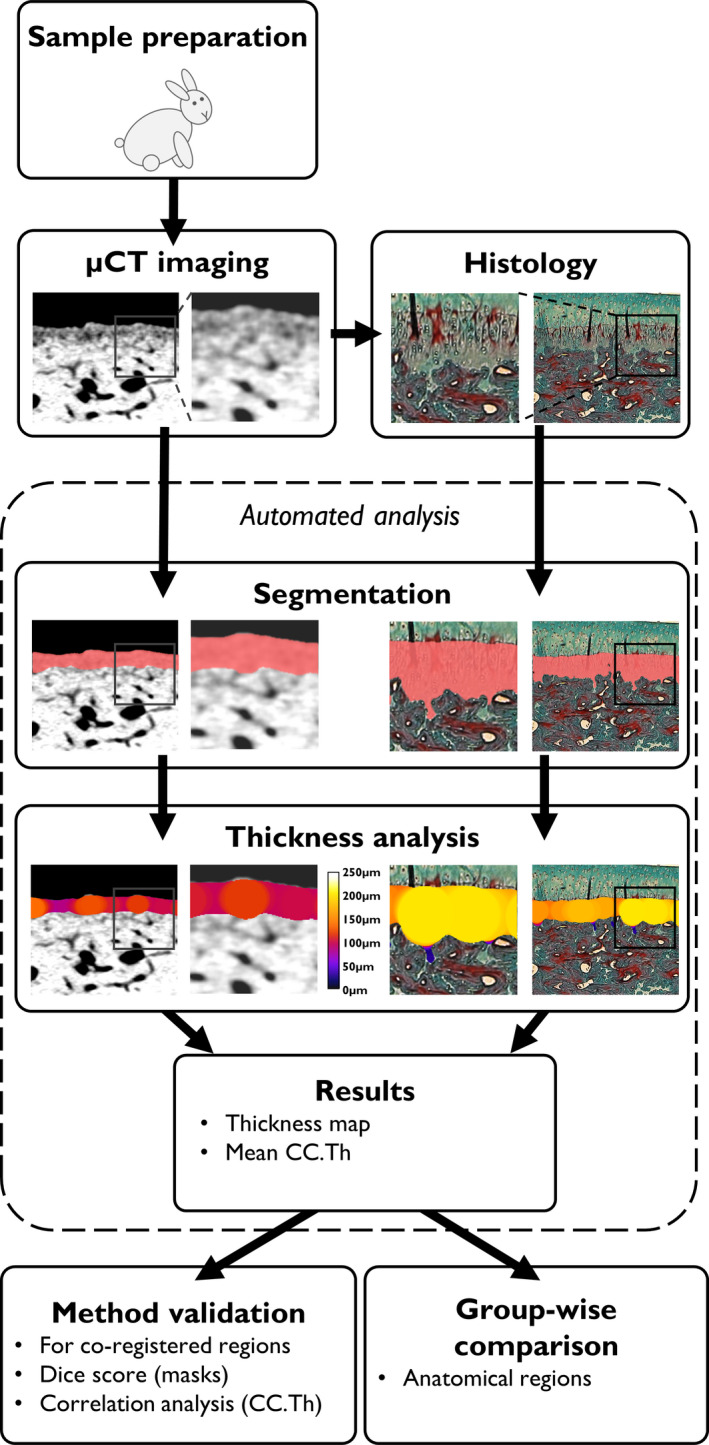
A flowchart summarizing the present study. Example from the femoral groove is shown with magnified insets, to highlight the similarities and differences between histology and µCT. After sample preparation, the tissue samples were imaged with µCT. Subsequently, the samples underwent histology processing, sectioning, and imaging with a light microscope. The preprocessing steps for the µCT data are illustrated in Figure S1. During the automated analysis process, the CC layer is predicted using the deep learning models, thickness analysis is conducted, and finally, quantitative parameters are estimated from the estimated thickness maps. The obtained values were used in the validation of the methods as well as for comparison between the anatomical regions of the knee. µCT, micro‐computed tomography; CC, calcified cartilage

The full analysis procedure of CC.Th is summarized in Figure 3. The thickness estimation of the CC layer was performed automatically using a Python‐based implementation of the local thickness algorithm. In the 2D case, the thickness assessment relies on mask skeletonization, a Euclidean distance transformation, and finally a simple circle‐fitting algorithm (Hildebrand & Rüegsegger, 1997). The 3D CC.Th analysis of the µCT volumes was conducted with a similar sphere‐fitting algorithm. From the estimated thickness maps, quantitative parameters such as mean‐, median‐, maximum CC.Th, or standard deviation of CC.Th can be calculated. In this study, we used the mean CC.Th as the quantitative parameter. The source code for the full segmentation and analysis procedure is published on our research unit's GitHub page (https://github.com/MIPT‐Oulu/RabbitCCS). For the µCT volumes, the thickness analysis took 2–3 h per sample (on a high‐end 12‐core CPU), whereas the analysis for the histology slices took roughly 3 s per image. For this study, the 3D thickness analysis was calculated with parallel processing on the Puhti supercomputer (https://research.csc.fi/csc‐s‐servers). This effectively reduced the computation time for the µCT volumes to roughly 6 min per sample.

To further investigate the applicability of the automatic segmentation on CC.Th analysis, a 2D analysis was performed between the manual segmentations and the out‐of‐fold predictions of the selected models. The thickness values were averaged for each sample with multiple histology sections or µCT slices.

### 2.6 Validation with histology

**FIGURE 4:**
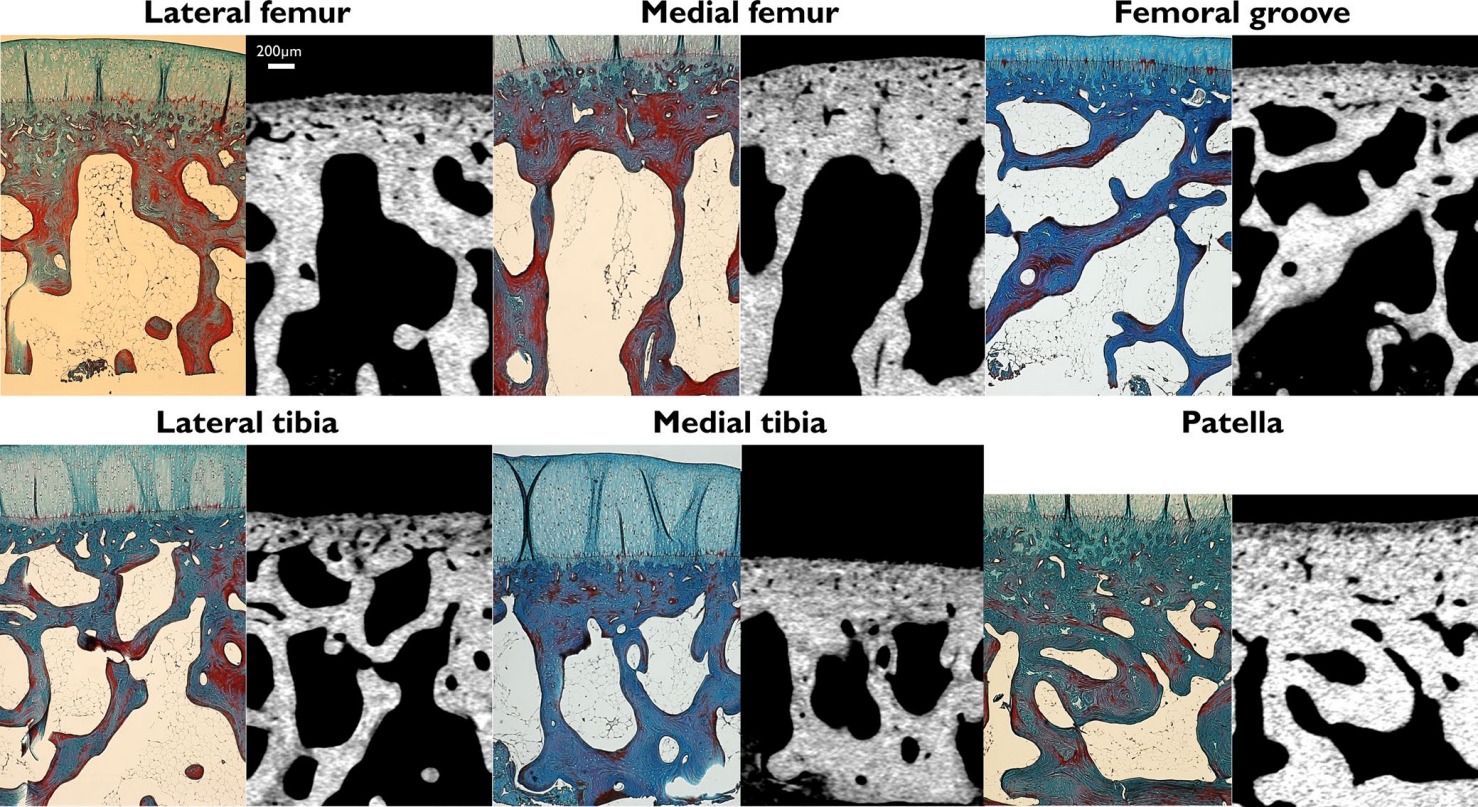
Examples from the co‐registered histology slices and µCT images. Scalebar for 200 µm is shown in the top left. The CC can be assessed using both imaging modalities, although the thinnest CC areas are not visible in the µCT images. Likely, these areas have a similar level of mineralization as the subchondral bone. The histology preparation could cause swelling of the tissue. This likely causes the largest proportional differences on the patella, which has a thick CC layer. µCT, micro‐computed tomography; CC, calcified cartilage

To compare the CC analysis between histology and µCT in 2D, matched µCT slices (Figure 4) were estimated using co‐registration based on rigid transformations with DataViewer (Bruker; version 1.5.2.4). A total of 24 samples (from four animals) were co‐registered with the corresponding histology sections to find the matching subchondral structures. As the search space is large when aligning the few micro meter thick histology sections with the full sample, the remaining samples in paraffin blocks were imaged again using the µCT scanner. The co‐registration of two µCT‐imaged samples is straightforward and allows for locating the cutting orientation and approximating the location of the histological sample. Final co‐registration was fine‐tuned by performing a second co‐registration between the original µCT datasets and the histology images. Five serial µCT images closest to the co‐registered histology image were selected. Finally, we calculated the CC.Th from the co‐registered histology image, whereas the CC.Th for µCT‐imaged samples was averaged from the five selected images.

### 2.7 Statistical analysis and performance evaluation

For the co‐registration experiment, a two‐tailed Pearson correlation and Bland–Altman analyses were conducted to compare CC.Th between the µCT and histology. The deep learning segmentation models were validated against the manual CC segmentations from µCT and histology using the Dice score. The thickness analyses using out‐of‐fold predictions and manual segmentations were compared using Pearson correlations. The anatomical differences of CC.Th were assessed using mean comparisons with Linear Mixed Effect Models (IBM SPSS Statistics; v.26), accounting for the rabbit ID as the random effect, and the anatomical location as the fixed effect. The significance was assessed with Least Significant Difference without Bonferroni correction.

## 3 RESULTS

### 3.1 Deep learning‐based segmentation

For both imaging modalities, the quality of the deep learning model predictions against the manual annotations (out‐of‐fold validation) is summarized in Figure 2 and Figure S2. By comparing the four different model architectures, ResNet‐34 with the U‐Net decoder yielded the highest mean Dice score for histology (Dice score = 0.891), whereas ResNet‐18 with FPN yielded the best performance for µCT segmentation (Dice score = 0.807). The quality of the segmentation on the full dataset was visually confirmed from virtual sections on orthogonal planes (Figure S4).

In addition, we compared the 2D CC.Th analysis for the manual and predicted CC segmentations for both modalities (Figure 2 bottom; Figure S5). With the selected model architecture, a high Pearson correlation was achieved between the manual and automatic CC.Th quantification from histology (*r* = 0.984, *p* < 0.001). The correlation between predicted CC.Th and manually segmented CC.Th in µCT images was also strong, although considerably smaller (*r* = 0.801, *p* < 0.001). This correlation analysis further supported the choice for model architecture (Figure 2, bottom).

### 3.2 Validation with histology

**FIGURE 5:**
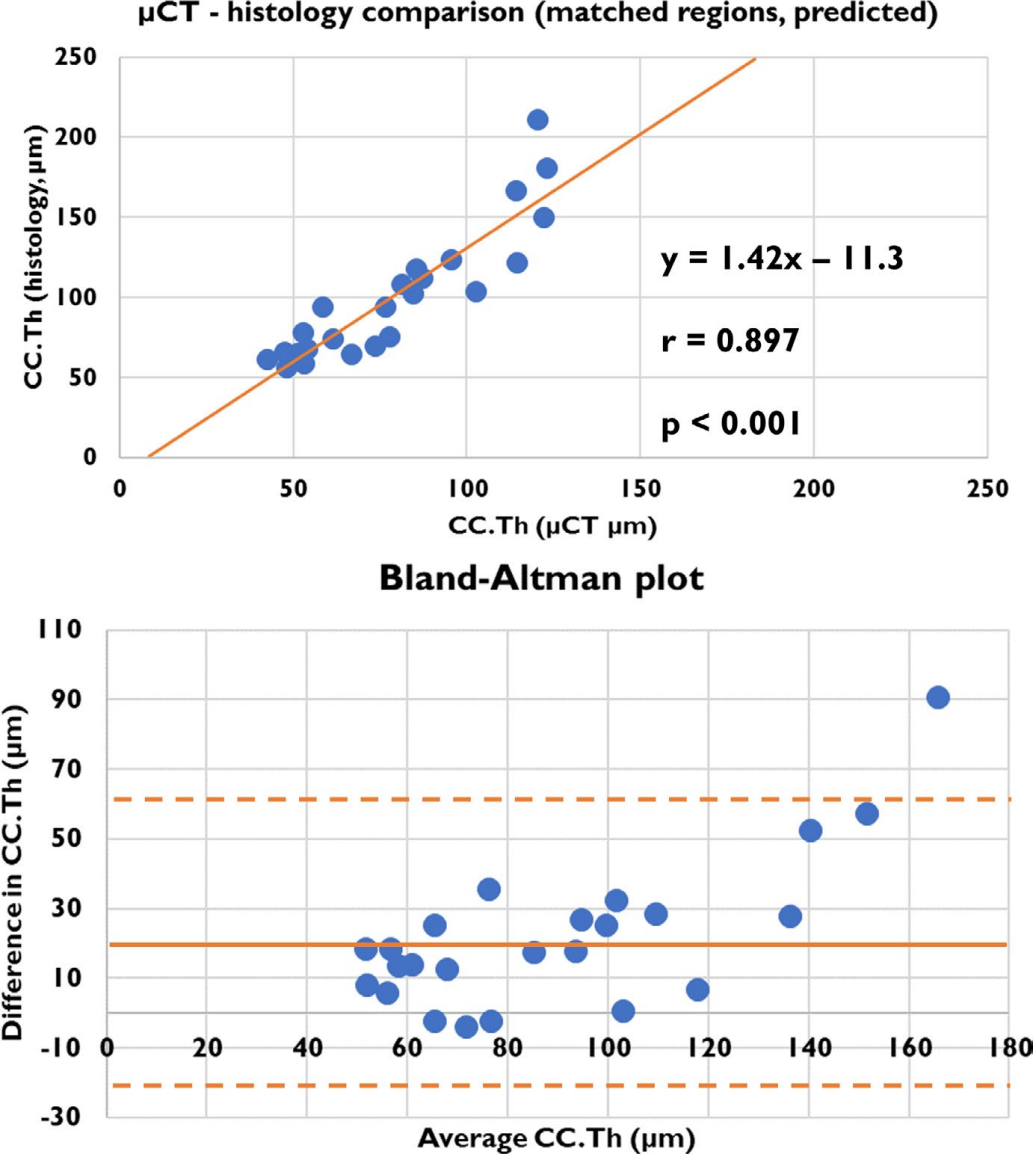
Quantitative CC.Th comparison of the matched histology and µCT regions based on automated segmentation. The equation for the linear fit, Pearson correlation and *p*‐value are shown in the top image. For the Bland–Altman plot, the bias is indicated with a horizontal line, and the distance of 1.96 standard deviations (95% CI) with a dashed line. The estimated values are highly correlated (*r* = 0.897) and the Bland–Altman analysis reveals that the µCT method yields 21.9 µm thinner CC.Th on average. The areas with a high CC.Th (mainly the patellar region) have the highest absolute differences between methods. µCT, micro‐computed tomography; CC, calcified cartilage

Examples of µCT images co‐registered with histology are shown in Figure 4. The results of the quantitative comparisons are shown in Figure 5 (predicted CC) and Figure S6 (manual segmentation). The automated µCT‐based measurements of CC.Th had a strong correlation (*r* = 0.897, *p* < 0.001) with a similar analysis on the co‐registered histology images. Furthermore, the µCT analysis had a good agreement (bias = 21.9 µm, standard deviation = 21.5 µm) with histology, based on the Bland–Altman analysis. However, the residuals were not normally distributed, due to larger differences in the patellar region. Furthermore, one of the patella samples yielded a larger difference than the 95% limit. Manual segmentation yielded a smaller correlation (*r* = 0.852, *p* < 0.001) as well as greater bias (36.9 µm) and standard deviation (30.9 µm) than the comparison using predicted masks. This time, two patella samples resulted in a difference outside the 95% limits of agreement.

### 3.3 Anatomical locations

**FIGURE 6:**
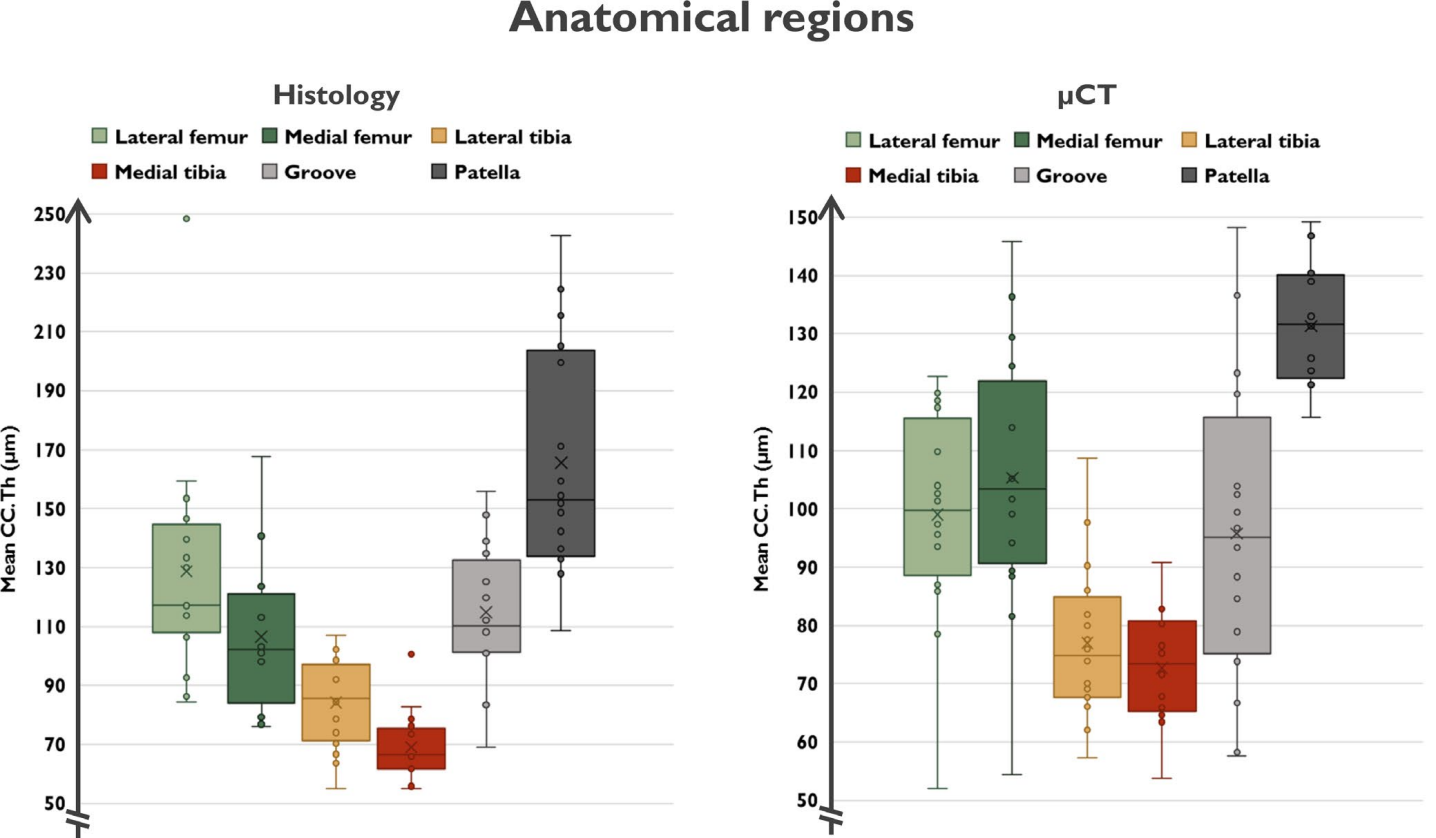
Boxplots illustrating the group‐wise CC.Th values obtained from the histology and µCT modalities. The median value for each group is shown with the horizontal line and mean value with the cross. From the graph, the anatomical regions can be divided into three categories: thin CC (lateral and medial tibia), intermediate CC (lateral and medial femoral condyles, femoral groove) and thick CC (patella). µCT, micro‐computed tomography; CC, calcified cartilage

An example of a thickness map and VOI inside a lateral plateau sample is shown in the Video S1. The differences in CC.Th based on anatomical variability are illustrated in Figure 6. According to the Linear Mixed Effects Model analysis on the histology and µCT results (Table 2), the mean CC.Th varies greatly between the studied anatomical regions (*p* < 0.001). The thickest CC was in the patellar region, whereas the thinnest CC was in the tibial regions (lateral and medial plateau). The histology analysis allowed for further separation of the lateral and medial femoral condyles (*p* = 0.026). Although the absolute differences in CC.Th were larger using histology analysis than with the µCT approach, the µCT results had a smaller variance for individual regions than that observed with histology, allowing for separation of the anatomical locations.

**TABLE 2:** Anatomical variation. Mean differences of mean calcified cartilage thickness between the six regions (in µm): Lateral (LF) and medial (MF) femoral condyle, lateral (LP) and medial (MP) tibial plateau, femoral groove (G) and patella (P). The differences were assessed using a Linear Mixed Effects Model analysis, with Least Significant Difference. Statistically significant differences (*p* < 0.05) are bolded. Detailed *p*‐values are shown for *p* ≥ 0.001

	MF	LP	MP	G	P
Histology					
LF	**22.2** ***p* = 0.026**	**44.5** ***	**59.7** ***	13.9 *p* = 0.162	**−36.9** ***
MF		**22.2** ***p* = 0.026**	**37.4** ***	−8.4 *p* = 0.397	**−59.1** ***
LP			15.2 *p* = 0.126	**−30.6** ***p* = 0.002**	**−81.4** ***
MP				**−45.8** ***	**−96.6** ***
G					**−50.8** ***
µCT					
LF	−6.3 *p* = 0.317	**22.1** ***p* = 0.001**	**26.3** ***	3.3 *p* = 0.604	**−32.3** ***
MF		**28.4** ***	**32.6** ***	9.6 *p* = 0.131	**−26.0** ***
LP			4.2 *p* = 0.503	**−18.8** ***p* = 0.004**	**−54.4** ***
MP				**−23.0** ***	**−58.6** ***
G					**−35.6** ***

Abbreviation: µCT, micro‐computed tomography.

*** *p* < 0.001.

## 4 DISCUSSION

Morphological analysis of CC may reveal novel understanding of musculoskeletal physiology and pathology. A suitable tool for structural analysis of CC would be µCT; however, the separation between bone and CC is extremely challenging. In this study, we developed a µCT‐based framework for 3D analysis of CC morphology. The framework utilizes state‐of‐the‐art deep learning segmentation for automated analysis of CC.Th. Finally, we compared CC morphology on different locations within the healthy rabbit knees. Our results demonstrate that CC.Th can be quantified not only from histology but also from µCT, which is feasible and efficient due to an automatic segmentation approach. The proposed method enables studying the 3D morphology of the mineralized CC without the time‐consuming and destructive histological processing and with minimal user‐induced bias.

Our results revealed that different CNN architectures were best suited for CC segmentation from histology and µCT images (Figure 2; Figure S2). The FPN decoder is computationally more efficient, but it introduces an up‐sampling layer for the model output. As a result, U‐Net provides more detailed predictions because the CC is predicted without a subsequent interpolation. The results show that the U‐Net decoder provided a slight advantage for segmenting the more complex CC structures in histology images. In the µCT images, such details are not visible, and FPN decoder yielded better results than the U‐Net one. Encoder‐wise, the deeper ResNet‐34 might yield even better performance than the ResNet‐18 encoder (He et al., 2016). However, the ResNet‐18 encoder with fewer layers than ResNet‐34 performed better on the µCT data than ResNet‐34. Thus, we suspect that the more complex ResNet‐34 may overfit when images become ambiguous, as in the case of the µCT images.

The automated CC segmentation performed particularly well for the histology samples (Figure S5). A relatively high Dice score coefficient (0.891) and similar CC.Th results compared with the manual annotations (*r* = 0.984) suggest that the automated and manual methods give virtually identical results. For the µCT data, the performance was weaker than for the histology data (Dice = 0.807, *r* = 0.801). However, the segmentation of CC from the µCT images is much more difficult than segmentation from histology slides. Therefore, this result was expected. Based on our experience, there is also a significant variation in manual CC segmentation between human annotators. However, when comparing the estimated 2D CC.Th between histology and µCT for co‐registered regions, there was strong agreement (*r* = 0.897). Although not explicitly shown in this study, we note that the proposed segmentation method generalizes fairly well, and could potentially be used to predict CC structures in diseased samples. This is supported by our initial experiments on osteoarthritic CC, and we aim to characterize both healthy and diseased CC in the future. Furthermore, one could also include manual annotations of diseased structures in the training data to increase segmentation performance.

We have previously shown that the subchondral bone plate imaged with µCT contains also the CC layer (Finnilä et al., 2017). Consequently, automated labelling of the CC layer could identify the true subchondral bone tissue accurately. The proposed method requires high‐resolution for resolving the mineralized cartilage. We believe that this is of high interest for studies that focus on the subtle changes in the bone plate, such as thinning due to increased remodelling. Such thinning of the bone plate has been suggested to occur already in the early stages of OA (Burr & Gallant, 2012).

The CC.Th measured from histology was on average 21.9 µm thicker compared with µCT, with highest differences on thickest regions such as the patella (Figure 5). Based on our results, the main differences are in the deep layers of CC which are only observed in histology images. We hypothesize that the less mineralized CC measured with µCT accounts for ‘young’ tissue, which is active and recently mineralized, and has distinct attenuation properties compared with the CC at the bone formation front and the bone tissue itself. Lower mineralization (hydroxyapatite content) of CC compared with bone has previously been reported using X‐ray diffraction (Rey et al., 1991; Zhang et al., 2012). However, multiple research groups have reported a higher mineralization of CC in backscattered electron imaging (Burr, 2004; Ferguson et al., 2003; Gupta et al., 2005) and Raman microscopy (Das Gupta et al., 2020) studies, at least for human tissue. Thus, we considered that there might be a possible contribution of partial volume effects related to cellularity. Most of the cellular structures are visible; however, the observed changes are likely related to tissue mineralization. On the other hand, the deep CC appears more mineralized with similar attenuation properties as the subchondral bone, making it impossible to identify it solely based on X‐ray methods. The samples with high CC.Th likely contain large, partly ossified areas of deep CC (such as the lateral condyle in Figure 1 and patellar region in Figure 4), leading to differences in CC.Th between the imaging methods. The initial, often extremely thin, CC layer could even have similar attenuation to bone before ossification. This would suggest that the µCT analysis specifically targets the newly mineralized CC. Therefore, we propose that our method could provide novel 3D information on tidemark advancement and other dynamic processes in CC. Due to the easier and non‐destructive sample preparation, the method might be preferable to standard histology for analyzing subtle changes of cartilage mineralization when combined with other bone analysis. In future studies, the method should be further developed to better understand the transition of CC to subchondral bone at the ossification front.

Interestingly, CC.Th depends greatly on anatomical location, as identified with both imaging methods. This is also consistent with our hypothesis. In the patellar region, CC.Th was the thickest among all locations of the rabbit knee. Femoral regions had intermediate CC.Th, whereas the thinnest regions were found in the medial tibial plateau region. We hypothesize that these variations in CC.Th are due to the distinct biomechanical environment in the different regions. First, the tibial plateau predominantly experiences compressive load due to body weight, whereas the patella experiences mainly shear forces that arise from the sliding joint articulation. Second, in the femur, the environment is a mix of these phenomena, i.e. the femoral condyles experience more compressive stress compared with higher shear forces on the groove. However, we did not find statistically significant differences in CC.Th between the condyles and groove. Finally, the higher shear stress experienced by the patella and femoral groove likely requires a stronger connection between the articular cartilage and the underlying subchondral bone plate, thus, resulting in higher CC.Th. Other studies have shown that the CC.Th of rabbit knees increases when subjected to chronic compression and that the CC is thicker in the lateral compared to the medial knee compartment (Roemhildt et al., 2012).

This study has several limitations: First, the decalcification process required for preparation of the histology slides may cause structural alterations in the tissue, such as swelling of the CC. Second, the intensity gradient between CC and subchondral bone can be ambiguous. This is especially the case for ultra‐thin or non‐existent CC. An ambiguous interface may appear because of endochondral remodelling resulting in bony protrusions into CC. Third, although an acceptable performance was achieved, the amount of training data used for the deep learning segmentation was relatively low. Examples from a greater number of animals may give a better performance, especially in the case of the challenging µCT segmentation. Fourth, our automated thickness analysis method is computationally expensive and does not scale well for large volumes. For routine use, more advanced scalable algorithms should be implemented, for example utilizing a distance ridge calculation (Dougherty & Kunzelmann, 2007). Fifth, the segmentation models might require fine‐tuning to data acquired from a different microscope or µCT scanner to ensure sufficient performance on new samples. In the future, more detailed CC structure could potentially be extracted by combining the presented approach with contrast‐enhancement (Kerckhofs et al., 2018; Nieminen et al., 2015) and/or imaging with devices capable of submicron resolution (Akhter et al., 2017). Finally, the proposed histology segmentation does not account for multiple tidemarks. Some evidence for tidemark duplication was found in few samples especially on medial femoral condyle, but the duplicated tidemarks were only faintly highlighted. We believe that the lack of duplicate tidemarks might be mainly due to the fact that we studied healthy rabbits but could also be caused by the properties of the chosen histological stain.

In conclusion, we have presented a promising method for the morphological analysis of CC with µCT. To the best of our knowledge, this is the first automated method for quantitative 3D analysis of CC.Th that has been sufficiently validated against the histological gold standard. It is a relatively simple extension to current µCT pipelines that allow 3D analysis of CC morphology. As a proof of concept, we could detect anatomical variation in the rabbit knee; the patellar region has the thickest CC and the tibial plateau region the thinnest. This structural difference between regions is presumably related to the diverse biomechanical environments, and thus the different requirements of the joint surfaces in different regions of the knee. Combined with other bone analysis, µCT imaging could provide an efficient alternative to histology when studying dynamic processes of the osteochondral junction, such as the tidemark advancement or bone plate remodelling.

## AUTHOR CONTRIBUTION

Study conception and design: S.J.O.R., L.H., P.T., A.T., R.K.K., S.S., W.H., M.A.J.F. Data collection: L.H., P.T., R.K.K., W.H., M.A.J.F. Method development: S.J.O.R., A.T., E.P., S.S., M.A.J.F. Data analysis and interpretation: S.J.O.R., L.H., A.T., S.S., M.A.J.F. Drafting the manuscript: S.J.O.R., L.H., M.A.J.F. Critical revision and approving the final version of the manuscript: All authors. S.J.O.R. takes responsibility for the integrity of the work.

## Supporting information

Fig S1

Fig S2

Fig S3

Fig S4

Fig S5

Fig S6

Supplementary Material

Supplementary Material

## Data Availability

The data that support the findings of this study are available from the corresponding author upon reasonable request.
