# Supplementary figures and images for: Automated analysis of rabbit knee calcified cartilage morphology using micro‐computed tomography and deep learning
Source: J Anat. 2021 Mar 29;239(2):251–63. doi: 10.1111/joa.13435 (PMC8273618; doi:10.1111/joa.13435)

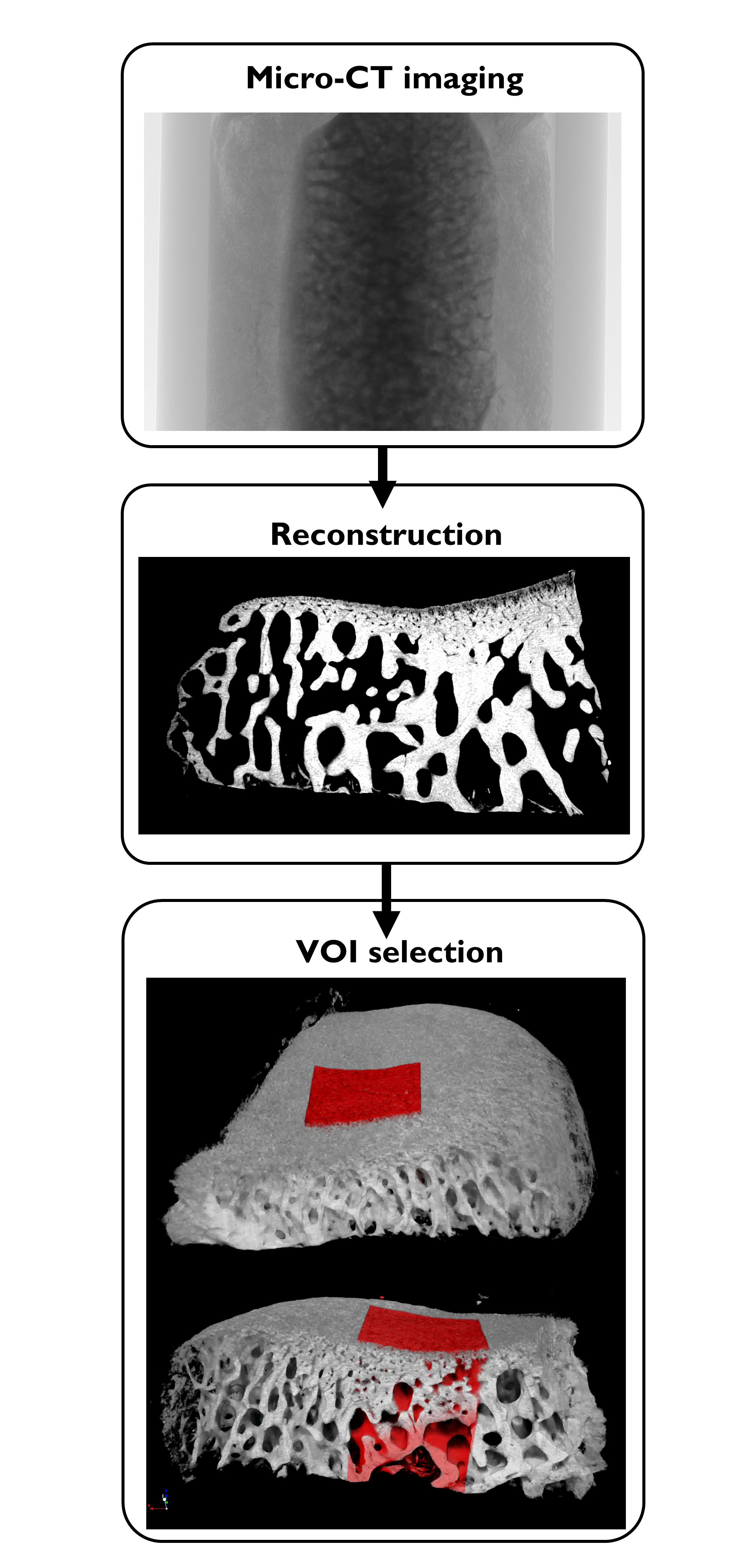

Supplement: Supplementary file 1 — Fig S1 [file JOA-239-251-s001.tif]

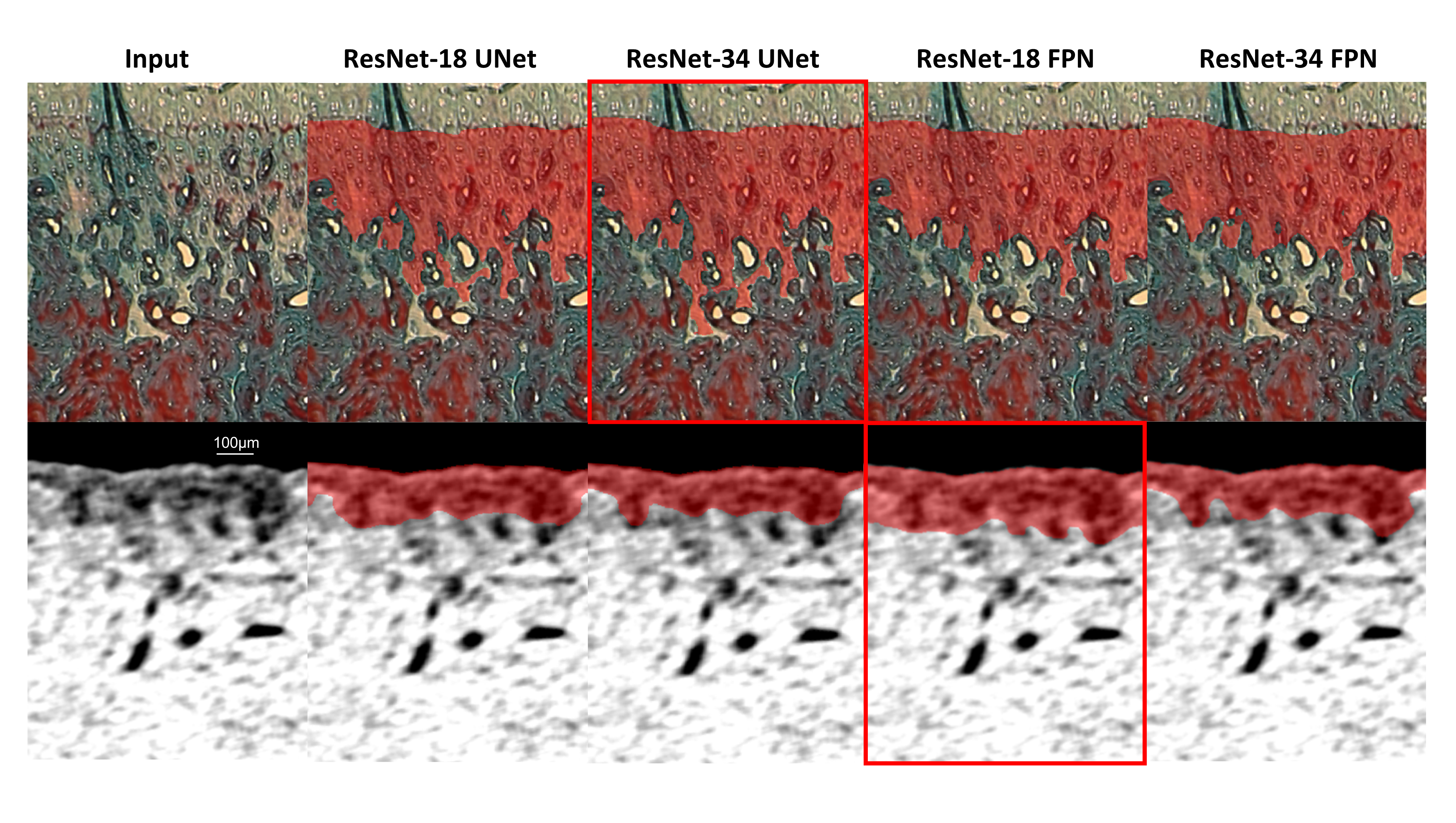

Supplement: Supplementary file 2 — Fig S2 [file JOA-239-251-s003.tif]

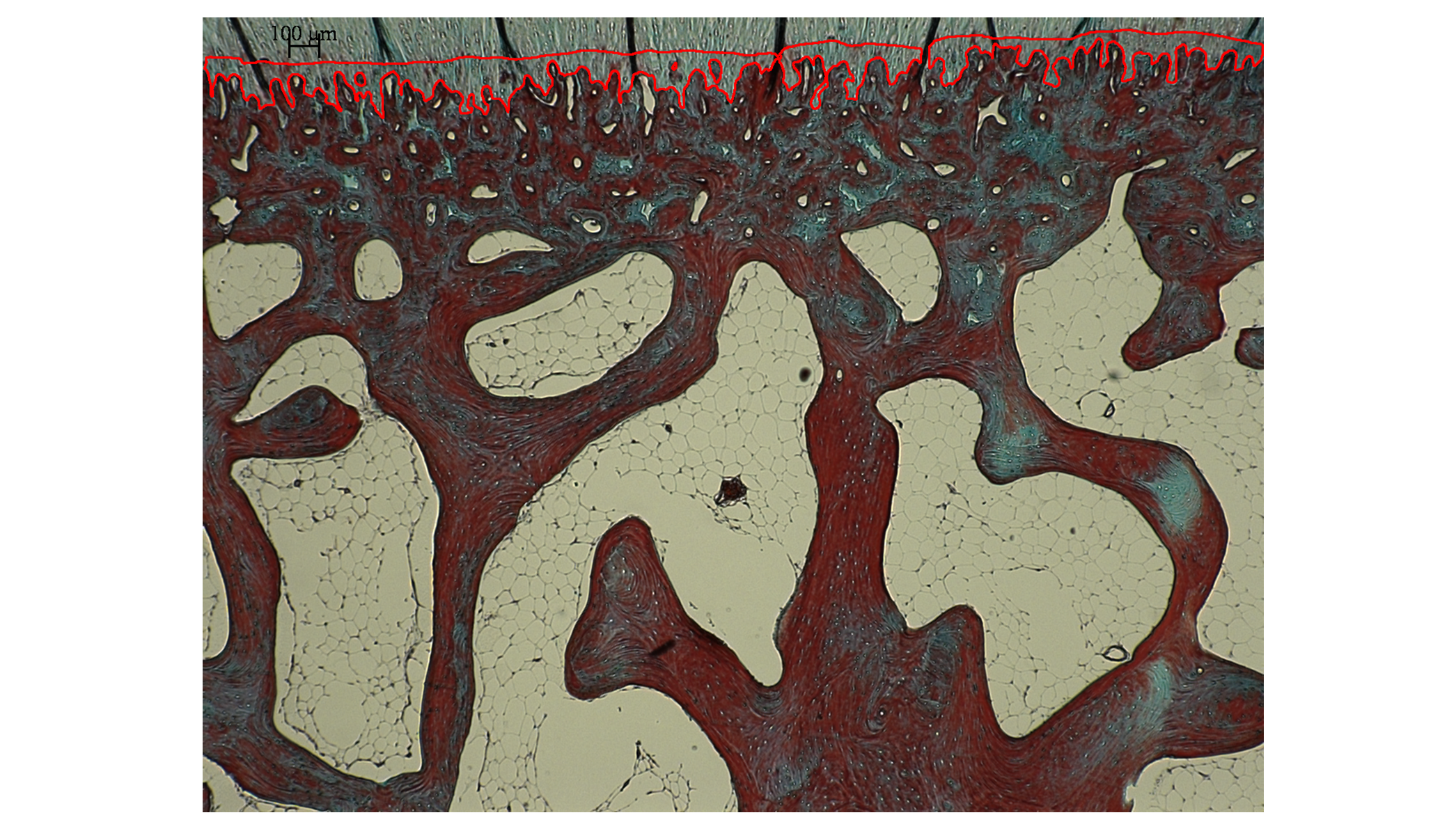

Supplement: Supplementary file 3 — Fig S3 [file JOA-239-251-s002.tif]

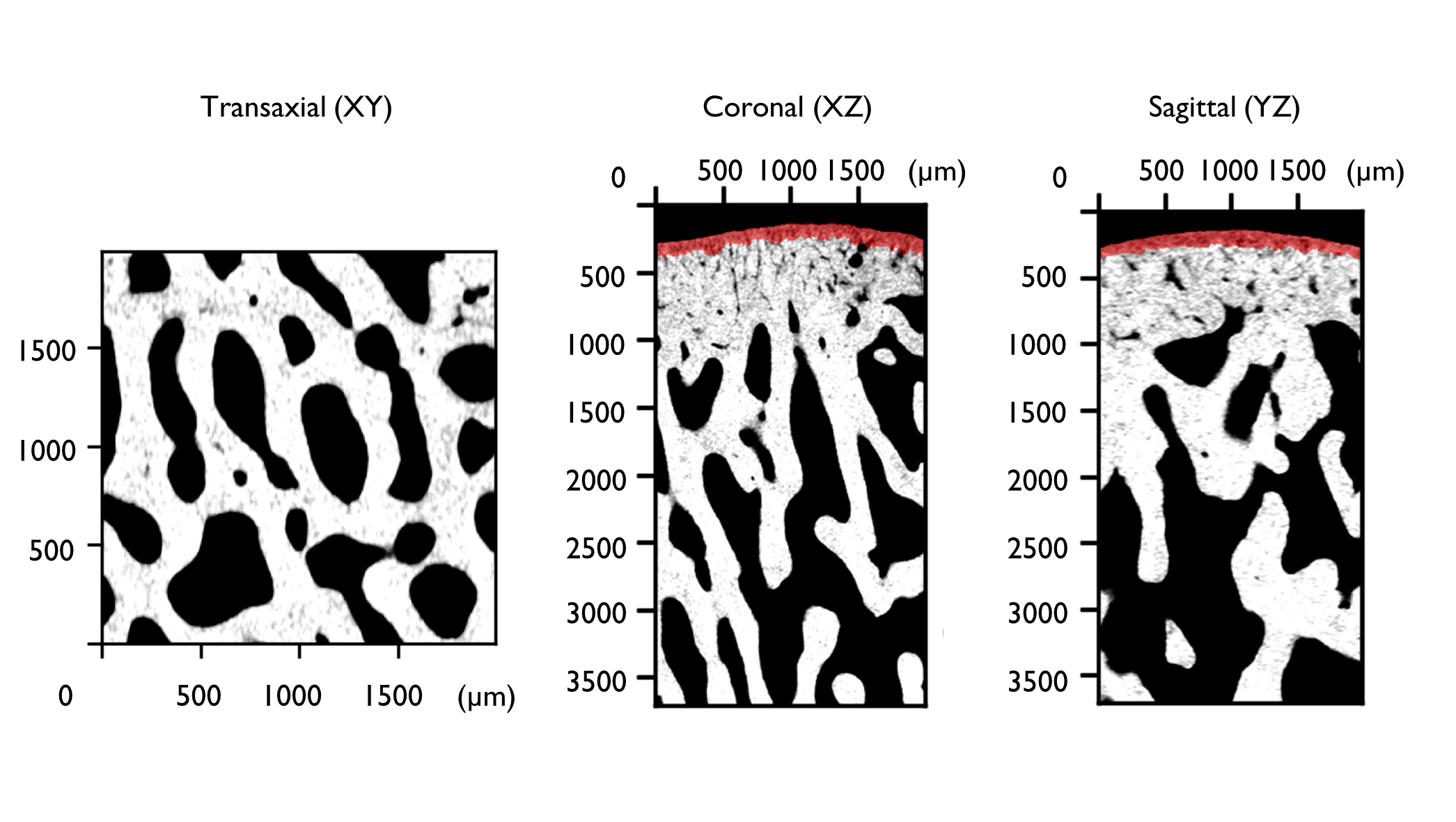

Supplement: Supplementary file 4 — Fig S4 [file JOA-239-251-s004.tif]

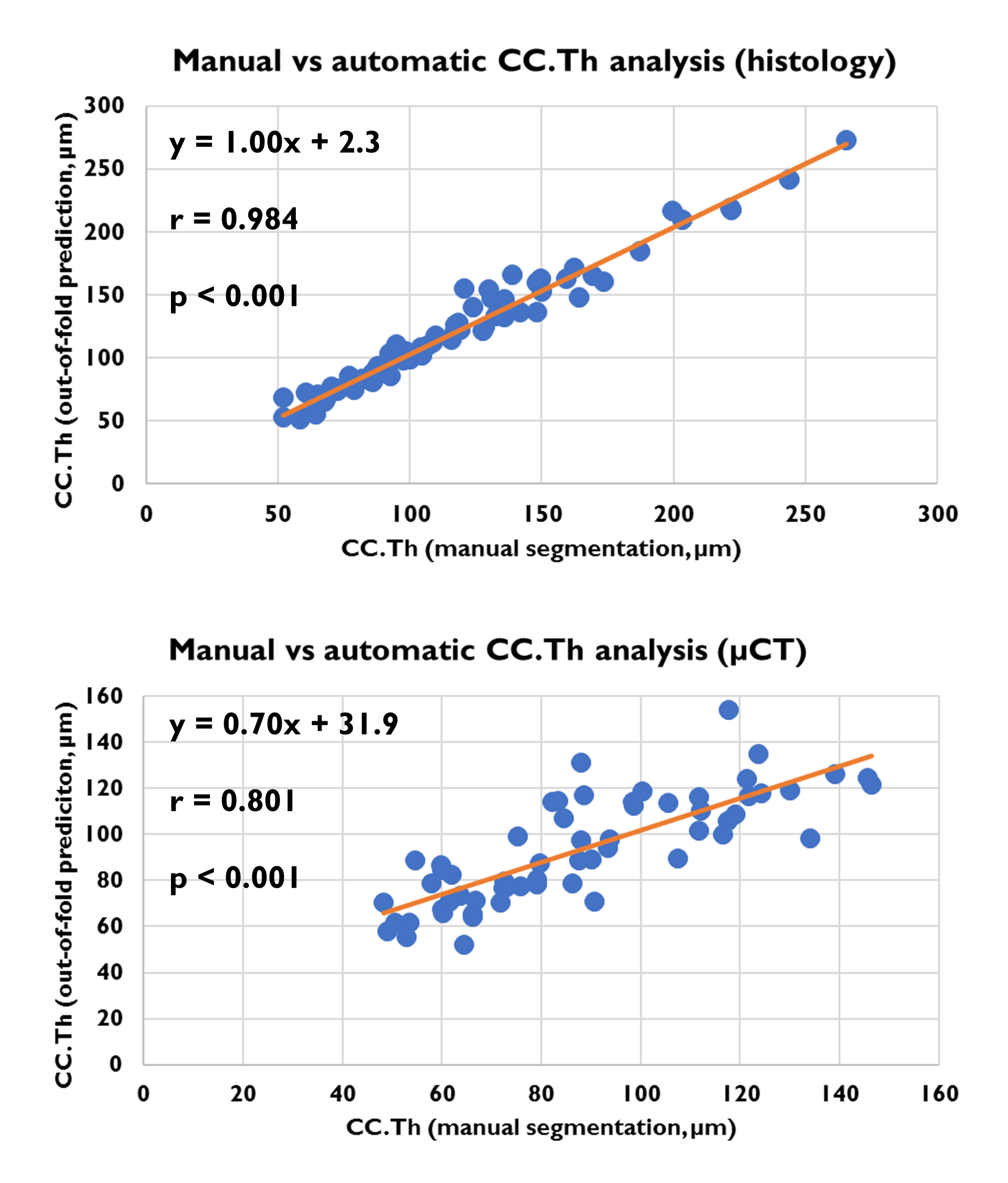

Supplement: Supplementary file 5 — Fig S5 [file JOA-239-251-s005.tif]

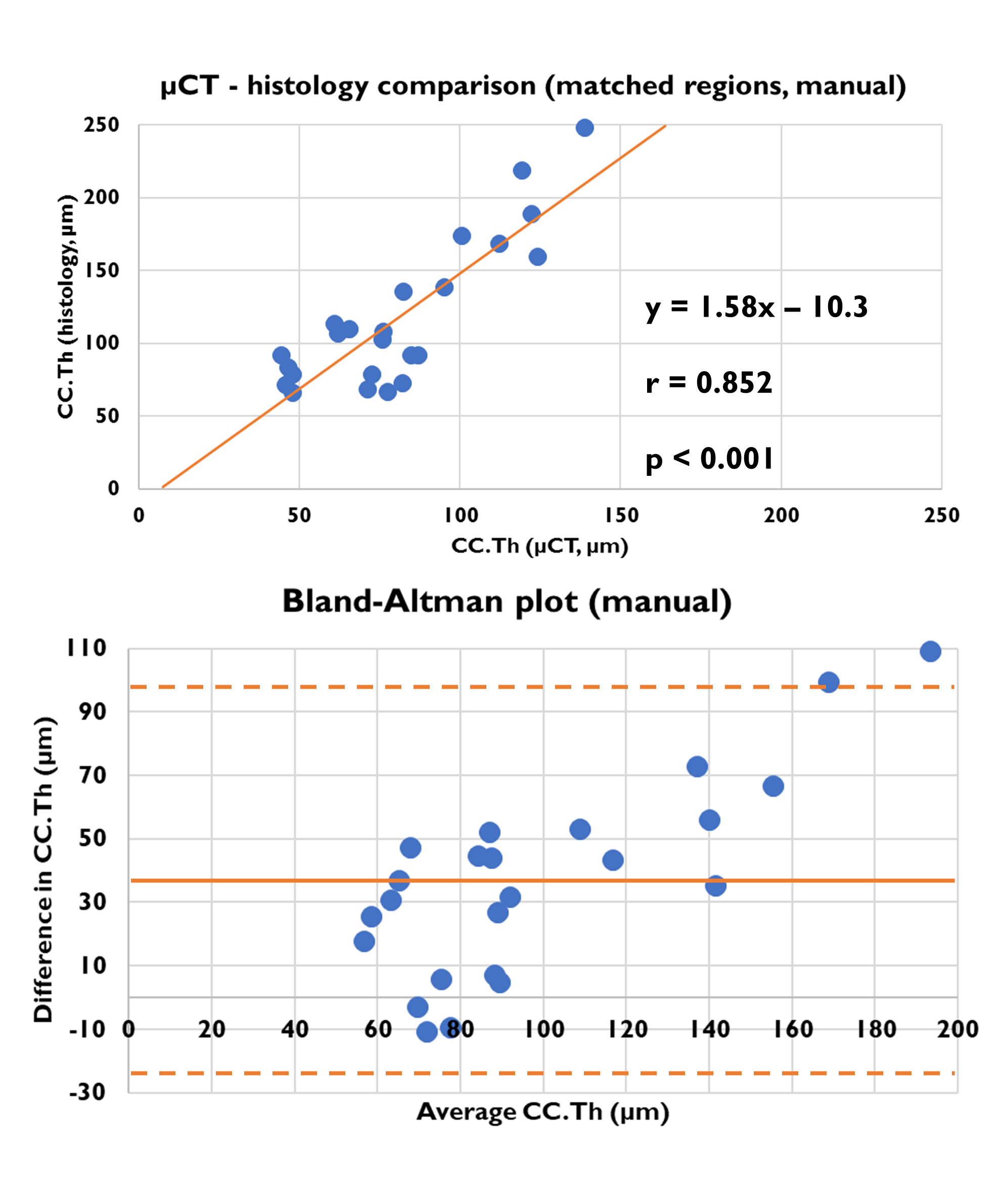

Supplement: Supplementary file 6 — Fig S6 [file JOA-239-251-s008.tif]
