# Supplementary material for: Automated analysis of rabbit knee calcified cartilage morphology using micro‐computed tomography and deep learning
Source: J Anat. 2021 Mar 29;239(2):251–63. doi: 10.1111/joa.13435 (PMC8273618; doi:10.1111/joa.13435)
Supplement: Supplementary file 7 — Supplementary Material [file JOA-239-251-s007.docx]

Automated analysis of rabbit knee calcified cartilage morphology using micro-computed tomography and deep learning

Santeri J. O. Rytky^1^, Lingwei Huang^2^, Petri Tanska^2^, Aleksei Tiulpin^1,3^^,4^, Egor Panfilov^1^, Walter Herzog^5^, Rami K. Korhonen^2^, Simo Saarakkala^1,3^, Mikko A. J. Finnilä^1,2,3^

^1^Research Unit of Medical Imaging, Physics and Technology, University of Oulu, Oulu, Finland;

^2^Department of Applied Physics, University of Eastern Finland, Kuopio, Finland;

^3^Department of Diagnostic Radiology, Oulu University Hospital, Oulu, Finland

^4^Ailean Technologies Oy, Oulu, Finland

^5^Human performance laboratory, Faculty of Kinesiology, University of Calgary, AB, Calgary, Canada

| **Supplementary Table 1.** List of random augmentations and the corresponding parameter ranges used in training the segmentation models. All augmentations are implemented using the SOLT library: <https://github.com/MIPT-Oulu/solt>. Since the µCT images were more challenging to label, we mainly used more subtle (smaller range) augmentations. | | | |
| --- | --- | --- | --- |
| **Random augmentation** | **Histology** | **µCT** | **Both** |
| Scale | [0.8, 1.2] | [0.9, 1.1] |  |
| Rotation (degrees) |  |  | [-10, 10] |
| Shear | - | [-0.1, 0.1] |  |
| Translation (pixels) | 50 | 30 |  |
| Flip |  |  | Horizontal |
| Crop (pixels) |  |  | [512, 1024] |
| Brightness | [30, 100] | [5, 15] |  |
| Contrast | 0.3 | 0.2 |  |
| Salt and pepper noise |  |  | 0.1 |
| Gaussian noise |  |  | 0.5 |
| Gaussian blur (kernel sizes, sigma) |  |  | ([3, 7, 11], [1, 5]) |
| Median blur (kernel sizes, sigma) |  |  | ([3, 7, 11], [1, 5]) |
| Gamma correction | [0.8, 1.2] | - |  |
| Color shift | [0, 255] | - |  |

| 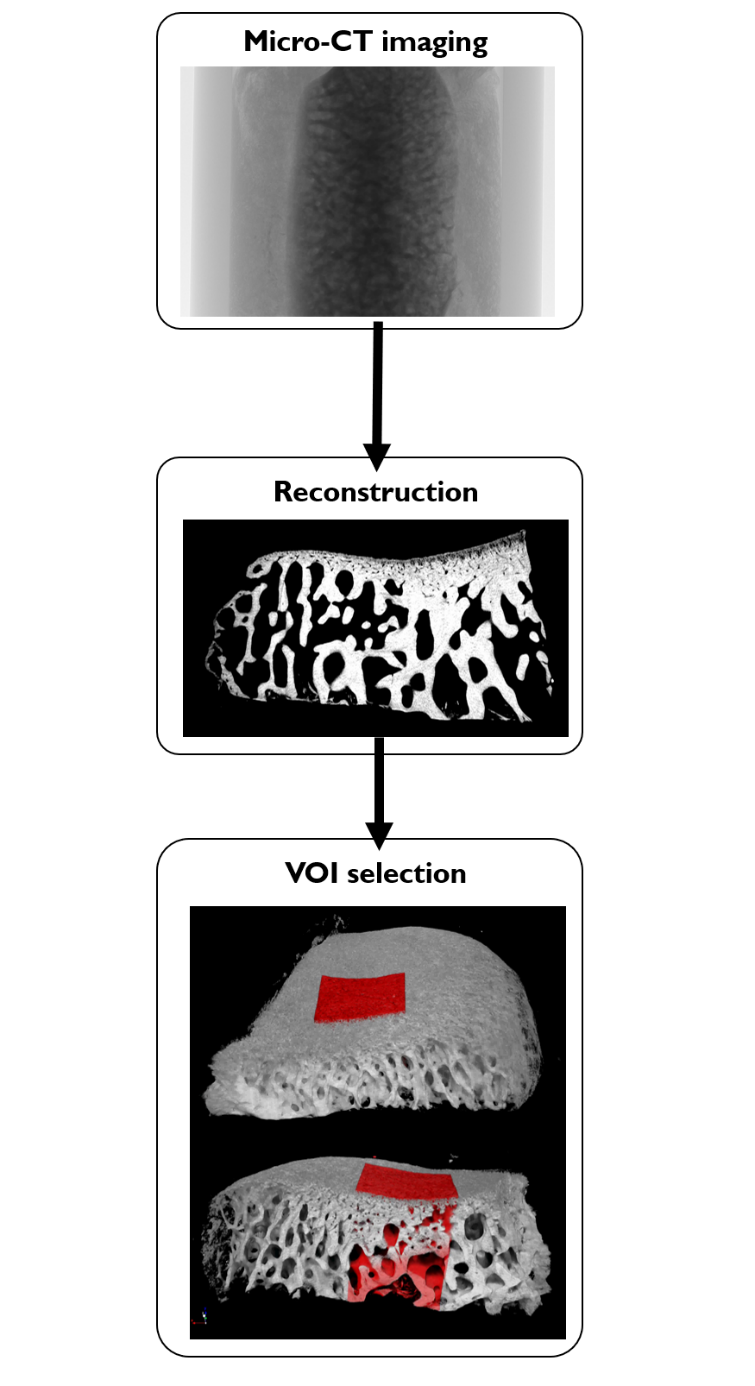 |
| --- |
| **Supplementary Figure 1.** The main preprocessing steps for the µCT data. In the top image, an example projection image is shown from a lateral tibial plateau sample. In the middle, a coronal section from the reconstruction result is displayed. The bottom image shows the 3D rendered sample with an example of the VOI selection. The top part shows an overview, while the bottom part includes a virtual section inside the sample. |
| \| 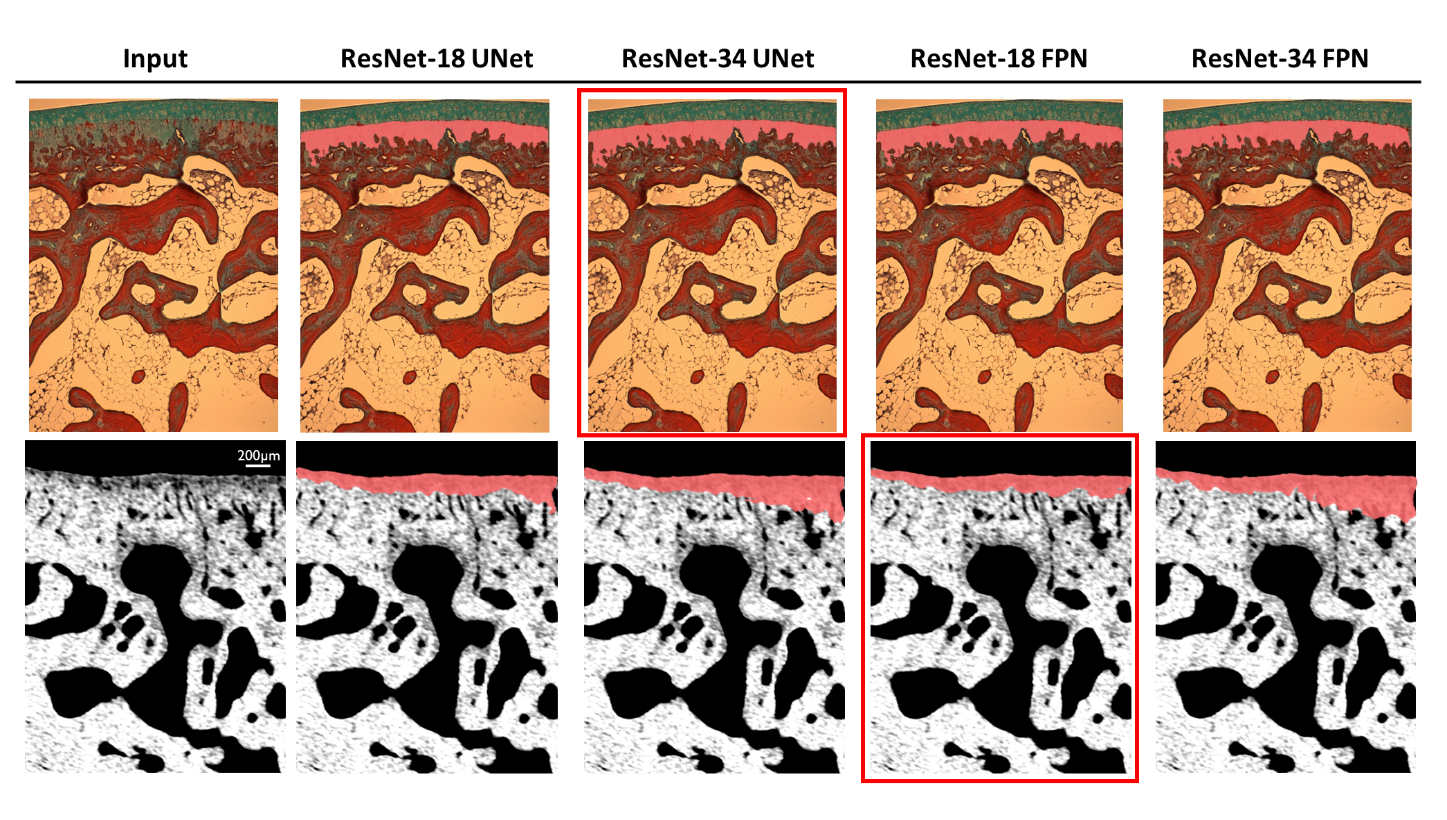 \| \| --- \| \| **Supplementary Figure 2.** Qualitative comparison of the predicted CC masks. The selected models are highlighted with a red rectangle. Since the FPN decoder requires upsampling of the segmentation result, small details of the histology masks are easily overlooked. This makes it better suited for the smooth µCT masks. U-Net preserves the complexity of the histology mask slightly better than FPN. For the µCT models, the complex ResNet-34 model may overfit the training data, thus yielding higher errors on the validation images. \| |
| \| **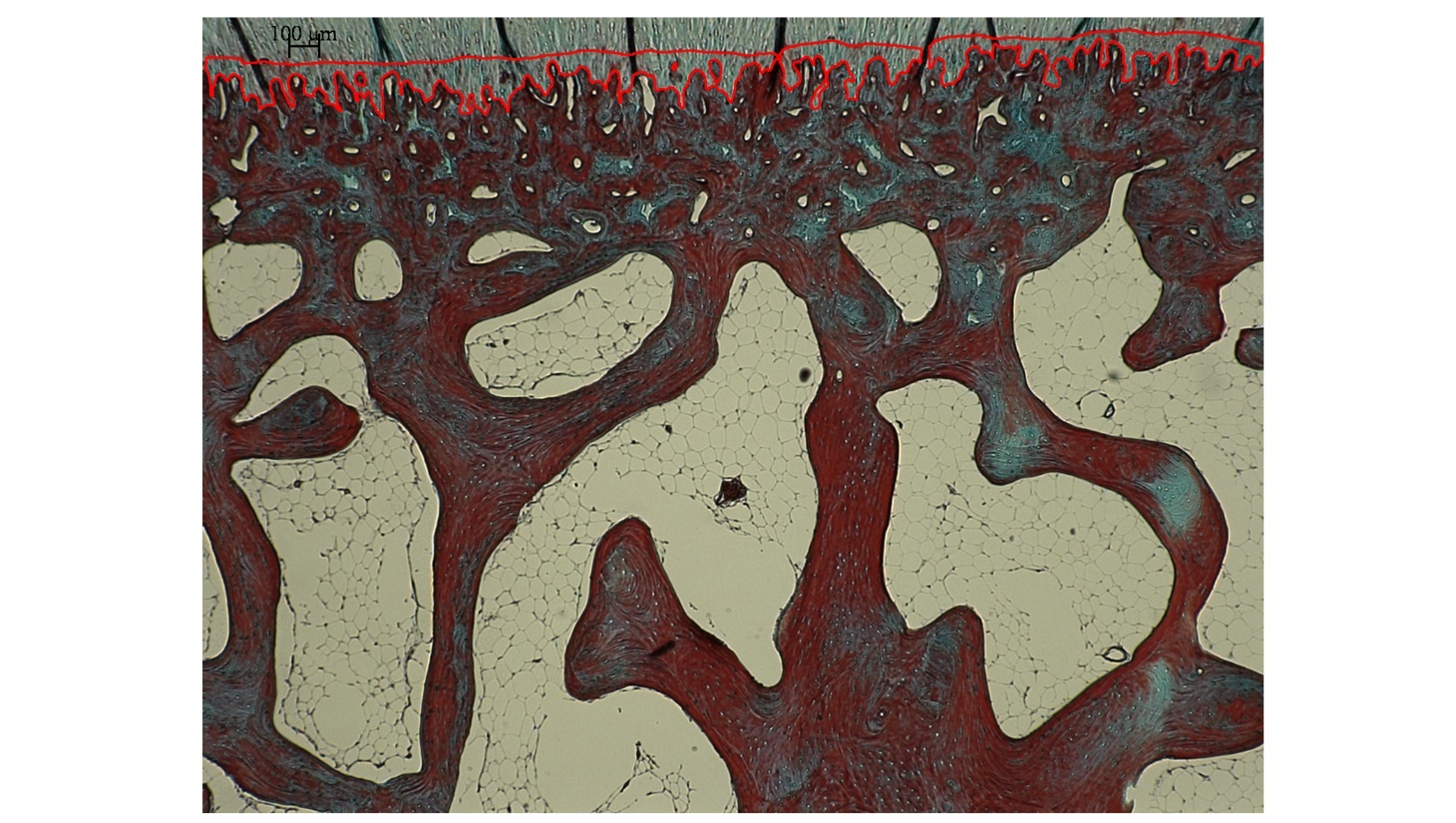** \| \| --- \| \| **Supplementary Figure 3.** Example histology slice with a disconnected prediction. The red outline illustrates the mask post-processed with the despeckle operation, removing areas smaller than 500 pixels. If only the largest mask would be retained, much of the real CC layer would be lost. \| |
| \| 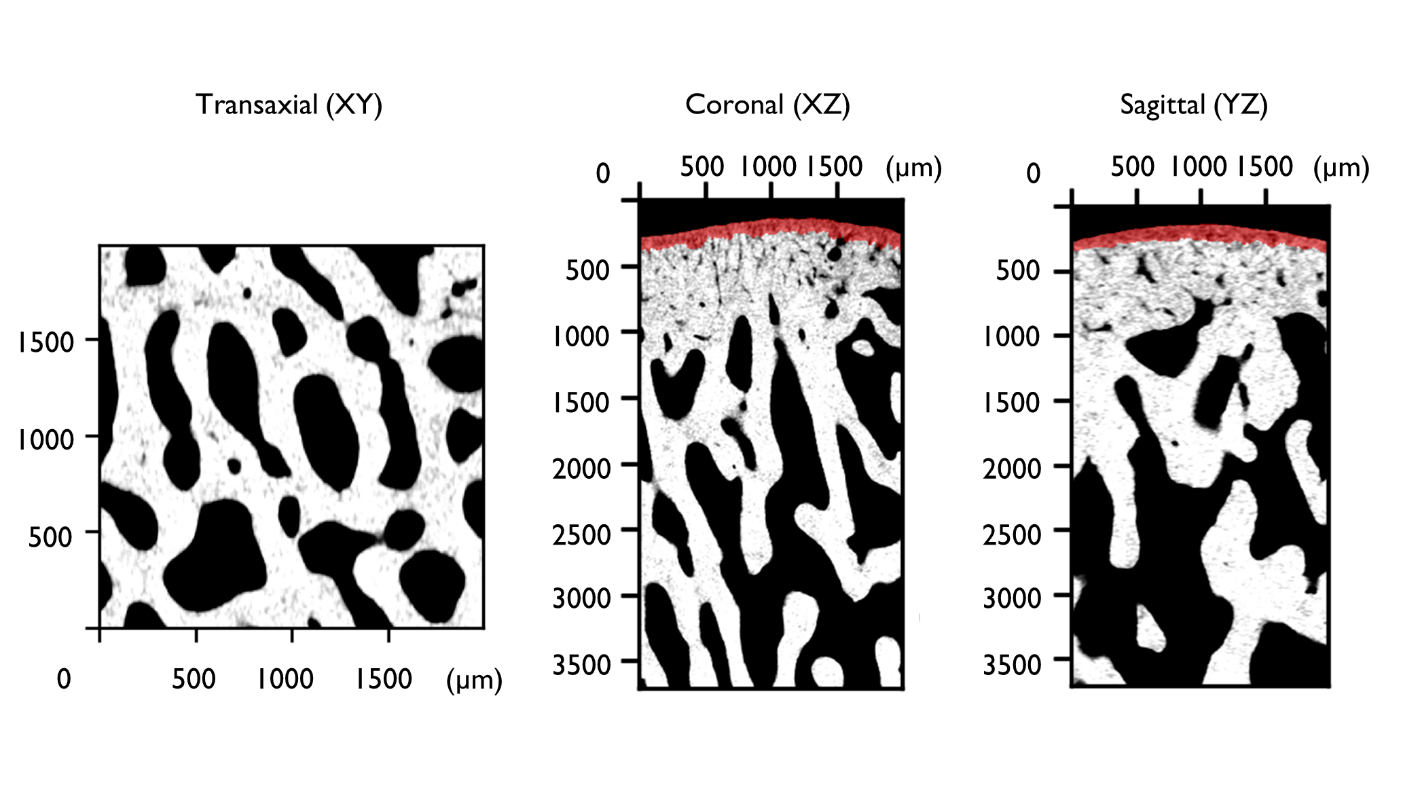 \| \| --- \| \| **Supplementary Figure 4.** Illustration for visual output of the predicted CC mask. A figure with three orthogonal planes on the sample is drawn after the inference, displaying the model output in red. \| |
| \| 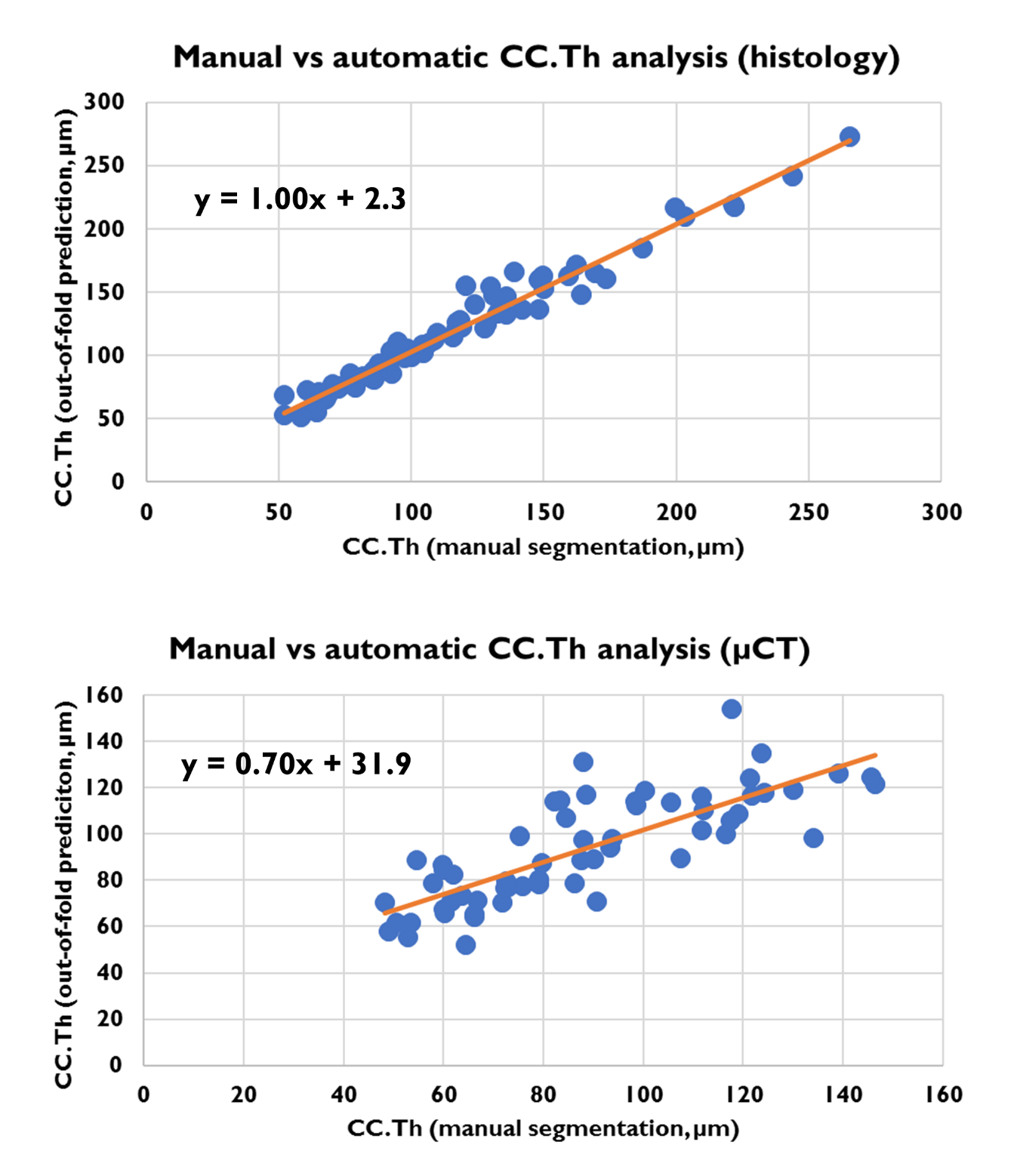 \| \| --- \| \| **Supplementary Figure 5.** Scatterplots for the CC.Th analysis based on manual and automatic segmentation. The equation for the linear fit is shown. With the histology images, the correspondence between the methods is extremely high. The more challenging µCT segmentation results in discrepancies between prediction and the gold standard. \| |
| \| 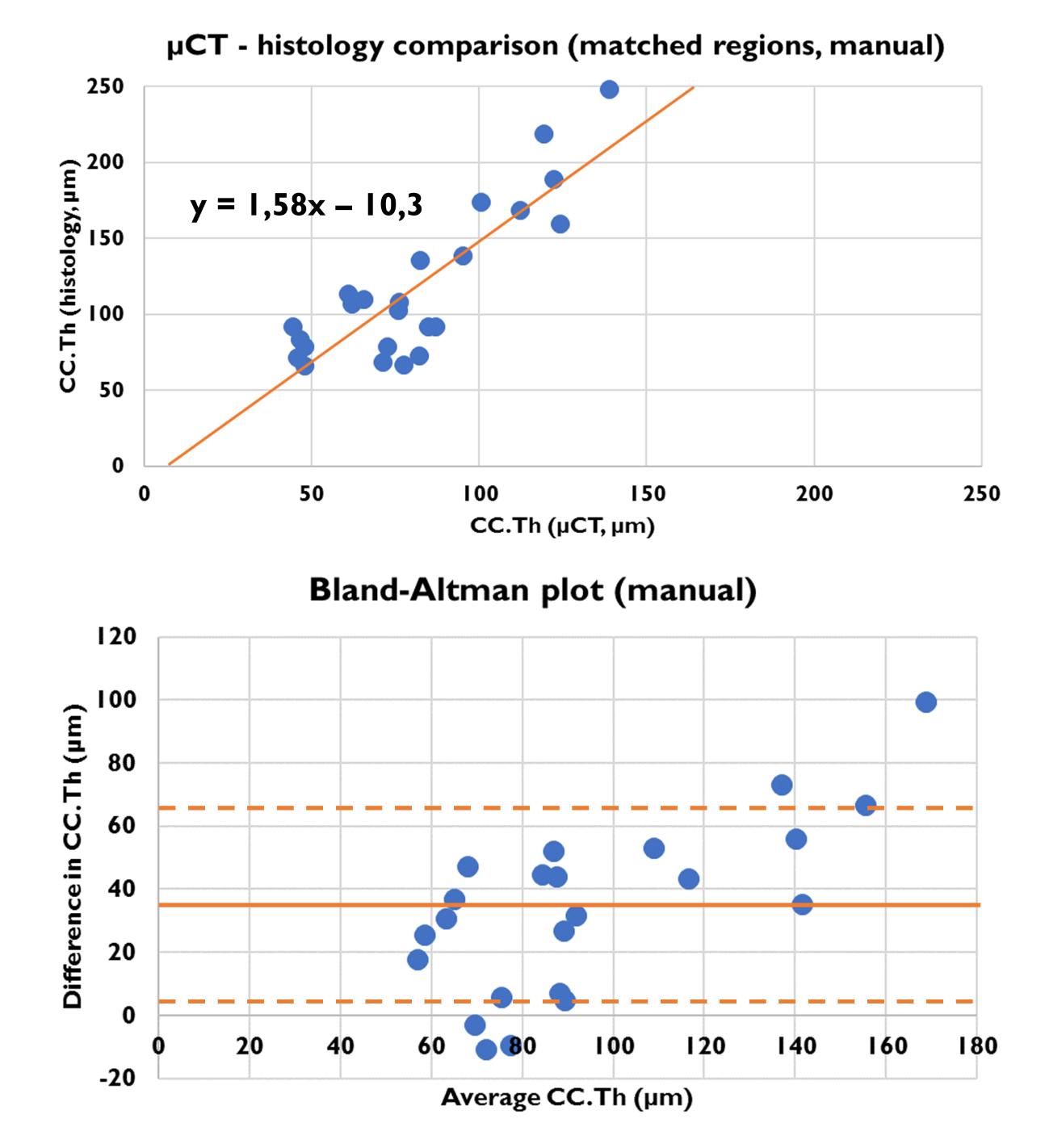 \| \| --- \| \| **Supplementary Figure 6.** Quantitative CC.Th comparison of the matched histology and µCT regions based on manual segmentation (compared to the predicted CC in Figure 5). The equation for the linear fit is shown in the top image. For the Bland-Altman plot, the bias is indicated with a horizontal line, and the distance of one standard deviation with a dashed line. The analysis yielded a Pearson correlation coefficient of 0.852, a bias of 36.9µm, and a standard deviation of 30.9µm. \| |

**Supplementary video.** An example of a thickness map and VOI inside a µCT-imaged lateral plateau sample for a healthy 12.5-month old rabbit is shown. The heatmap visualizes the thickest regions in white.
